# Coal seam gas industry methane emissions in the Surat Basin, Australia: comparing airborne measurements with inventories

**DOI:** 10.1098/rsta.2020.0458

**Published:** 2021-11-15

**Authors:** Bruno G. Neininger, Bryce F. J. Kelly, Jorg M. Hacker, Xinyi LU, Stefan Schwietzke

**Affiliations:** ^1^ MetAir AG, Airfield LSZN, Switzerland; ^2^ School of Biological, Earth and Environmental Sciences, UNSW Sydney, Sydney, New South Wales 2052, Australia; ^3^ Airborne Research Australia, Parafield Airport, South Australia 5106, Australia; ^4^ College of Science and Engineering, Flinders University, Adelaide, South Australia 5001, Australia; ^5^ Environmental Defense Fund, Third Floor, 41 Eastcheap, London EC3M 1DT, UK

**Keywords:** methane, coal seam gas, Surat Basin, Australia, airborne measurements, UNFCCC inventory, climate change

## Abstract

Coal seam gas (CSG) accounts for about one-quarter of natural gas production in Australia and rapidly increasing amounts globally. This is the first study worldwide using airborne measurement techniques to quantify methane (CH_4_) emissions from a producing CSG field: the Surat Basin, Queensland, Australia. Spatially resolved CH_4_ emissions were quantified from all major sources based on top-down (TD) and bottom-up (BU) approaches, the latter using Australia's UNFCCC reporting workflow. Based on our TD-validated BU inventory, CSG sources emit about 0.4% of the produced gas, comparable to onshore dry gas fields in the USA and The Netherlands, but substantially smaller than in other onshore regions, especially those where oil is co-produced (wet gas). The CSG CH_4_ emission per unit of gas production determined in this study is two to three times higher than existing inventories for the region. Our results indicate that the BU emission factors for feedlots and grazing cattle need review, possibly requiring an increase for Queensland's conditions. In some subregions, the BU estimate for gathering and boosting stations is potentially too high. The results from our iterative BU inventory process, which feeds into TD data, illustrate how global characterization of CH_4_ emissions could be improved by incorporating empirical TD verification surveys into national reporting.

This article is part of a discussion meeting issue ‘Rising methane: is warming feeding warming? (part 1)’.

## 1. Introduction

Methane (CH_4_) is the second most important anthropogenic greenhouse gas, contributing about 25% to the global warming experienced to date [1,2]. Global atmospheric CH_4_ mole fractions have increased from around 700 parts per billion (ppb) in the pre-industrial era [3] to around 1870 ppb in 2020 [4], increasing more than three times as fast as CO_2_ [5,6]. Fossil and microbial anthropogenic sources as well as natural CH_4_ sources may have acted in tandem to accelerate this increase in 2007 and 2014 [7].

Mitigation of anthropogenic CH_4_ emissions is considered an effective way to slow the rate of global warming in addition to CO_2_ emission reductions to minimize the warming magnitude [8]. Substantial emission cuts along the oil and gas (O&G) industry supply chain can be achieved at no net monetary cost [9], especially when also factoring in the social cost of CH_4_ [10]. In fact, governments around the world including Canada, Colombia, Mexico and the USA have already committed to substantial CH_4_ emission mitigation from the O&G industry supply chain in parallel to CH_4_ intensity targets set by that industry [11].

In addition to quantifying the total CH_4_ emission magnitudes, understanding their individual sources and mechanisms is key for developing effective mitigation strategies and for drafting adequate regulations as well as for validating whether reduction targets are in fact being met. Scientific methods exist, are being further developed and have been applied around the world to detect, locate and quantify emitters across the emission size distribution to support such mitigation [12,13].

One of the so-called unconventional gas production sources is coal seam gas (CSG), sometimes also labelled coalbed CH_4_. Complete global production data of CSG are usually not publicly available, but CSG accounted for about 5% of natural gas production in the USA throughout the 2010s [14], 24% of Australia's gas production in 2018 [15,16] and rapidly increasing amounts in China (the world's largest coal producer), for which reliable CSG production data are difficult to obtain [17]. Given the operational differences between CSG and other forms of O&G production (including dry and wet gas production from conventional and shale formations), it is essential to understand CH_4_ emissions from CSG production alone. This includes the fact that CSG fields require more gathering lines, pumps and compressors than conventional gas fields because (i) CSG wells are less productive (i.e. larger number of wells needed) and (ii) they produce gas at lower pressure [18].

CH_4_ emissions from CSG production are not well characterized as this source of the O&G supply chain is currently under-sampled in the peer-reviewed literature. Most published research has been carried out in the Surat Basin in Queensland, Australia, which produced 77% of Australia's CSG in 2018 [15,16]. The portion of the Surat Basin covered by the work presented here represents 57% of the Queensland Government gas production inventory. According to the Australian Government, about 0.5% of Australia's produced CH_4_ is emitted to the atmosphere [18,19]. Six previous ground surveys of CH_4_ emissions from the Surat Basin CSG fields are reviewed in [12,20]. These ground-based surveys focused on identifying point sources and analysing the chemistry of the CH_4_ emissions [21] measured 37 well pads from three participating operators who granted site access, which emitted on average 0.2 kg CH_4_ h^−1^. Later, Tsai *et al*. [22] surveyed 137 well pads measuring at most 0.4 kg CH_4_ h^−1^, although details of the survey and site selection procedure are not disclosed. Recently, total CSG-related CH_4_ emissions in the Surat Basin were quantified based on quasi-continuous measurements of CH_4_ carried out at two towers over an 18-month period [23]. A Bayesian inversion framework was used to attribute CH_4_ enhancements above background levels to different source regions and emission source categories. The study finds that CH_4_ emissions from CSG-dominated subregions are about one third greater than a bottom-up (BU) inventory estimate described in [23]. The authors of [23] acknowledge the dependence of their results on the choice of the Bayesian prior estimate, i.e. the BU inventory, and that more work is needed to improve the source attribution in the region. The BU inventory used in [23] is henceforth referred to as the Katestone inventory.

There are multiple challenges for quantifying CSG-related CH_4_ emissions in the Surat Basin. This includes the presence of large non-CSG CH_4_ emitters (ruminants in particular), the associated need for spatial separation of emission sources from multiple source categories and the distribution of emissions over a large area. Additionally, the small ethane content (less than 1%) of CSG [24,25] precludes the use of atmospheric ethane measurements as the basis for attributing total CH_4_ emissions into fossil (i.e. CSG) and non-fossil sources. Atmospheric ethane measurements have been used in multiple previous studies for source attribution [26–28], which required the quantification of total CH_4_ emissions only at the full O&G basin-level (instead of locating and quantifying individual O&G facilities or O&G-dominated subregions). We have demonstrated that there is potential to use both *δ*^13^C_CH_4__ and *δ*D_CH_4__ source signatures to separate CSG and cattle emissions from other CH_4_ sources in the region [20]. The use of isotopes for verifying inventories is discussed below.

Here, we present the results of the first intensive measurement campaign in the Surat Basin to quantify CH_4_ emissions comprehensively from all CSG and other sources using established surveying techniques. The objectives of this study are threefold. First, aerial measurements and multiple modelling techniques were employed to quantify total CH_4_ emissions from multiple subregions and individual CSG and non-CSG facilities of the Surat Basin in a top-down (TD) approach. Second, a new spatially resolved, BU emission inventory of all existing CH_4_ source categories was developed, using the latest publicly available data on emission source counts. The CSG and non-CSG categories quantified and emission factors used closely follow the Australian Government's national greenhouse gas inventory report workflow submitted to the United Nations Framework Convention on Climate Change (UNFCCC) on Australia's national greenhouse gas inventory for 2018 [29]. The spatially resolved UNSW CH_4_ emission inventory was then used to assist in the CH_4_ source attribution of the independently derived aerial-based total CH_4_ emission quantification. In this paper, we (i) derive the BU and empirical TD estimates, (ii) attempt to reconcile potential differences between the absolute TD and BU emission estimates of the CSG and non-CSG sources in the Surat Basin, (iii) compare the results with previous studies, and (iv) discuss the implications for improving Australia's UNFCCC CH_4_ emissions reporting.

## 2. Methods

**Figure 1.**
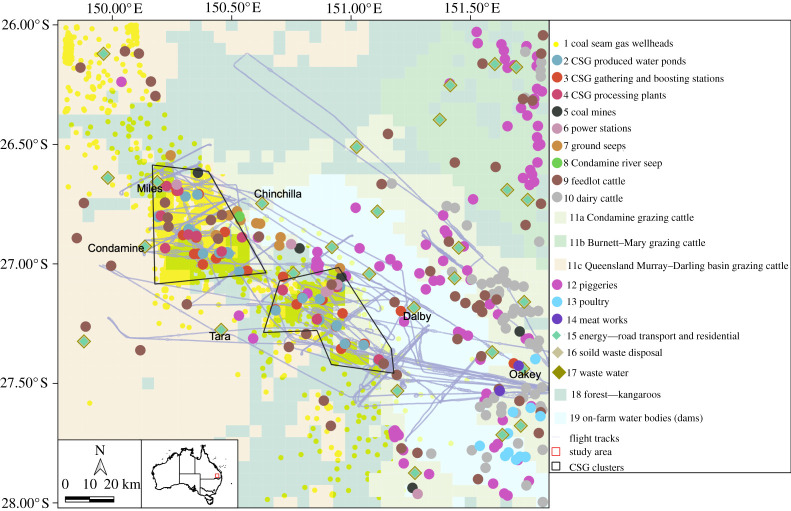
Distribution of point sources in the southeast portion of the Surat Basin CSG fields together with the flight tracks for all research flights, which cover the two CSG clusters described in the text (outlined with black lines). Details on the emission contributions from each source are provided in electronic supplementary material, tables ST1 and ST2. Refer to electronic supplementary material, figures SF7–SF12 for additional information on the flight tracks, and electronic supplementary material, figures SF1–SF3 for the sources displayed on satellite images. Within the map, the symbols for energy (15), solid waste (16) and waste water (17) are co-located. (Online version in colour.)

The main CSG-related sources surveyed in this study are located in an NW to SE trending region of approximately 50 km (NE–SW) by 150 km (NW–SE). Within this area, there are two main CSG clusters located between Condamine and Chinchilla, and Tara and Dalby (figure 1).

The known emission sources are comprehensively discussed in [20,23]. CH_4_ emissions from 25 sources were quantified (electronic supplementary material, table ST1) covering CSG, coal mining, agricultural, natural and residential emissions. The study area, the locations of the different emission sources and the flight tracks of the 11 research flights over seven days (10–21 September 2018) are shown in figure 1. The ‘study area’ is the whole area shown in figure 1, and the ‘TD domain’ is the portion of the study area that is covered by the aerial mass balance shown in figure 1, which includes the two CSG clusters. More details are presented in electronic supplementary material, figures SF1–SF3. Our TD–BU comparison (including the UNSW inventory and literature inventories) will refer to emissions only in the TD domain.

### (a) Development of a spatially resolved bottom-up inventory for 2018

For the study area defined above, we estimate spatially resolved CH_4_ emissions based on literature emission factors from known sources, including natural, CSG, coal mining, agricultural and residential categories. Herein this BU inventory is referred to as the UNSW inventory. The development of this inventory was needed because insufficient details of the existing Katestone inventory [23] are publicly available. We then use this gridded BU inventory to (i) attribute source categories to the airborne measurement-based TD estimates, and (ii) compare BU and TD total CH_4_ emissions. A comprehensive discussion of the BU calculation of the CSG-related CH_4_ emissions is presented in electronic supplementary material, tables ST1–ST3. Extensive details for the BU inventory primary datasets for the CSG sector, other industrial, agricultural and residential sources, along with the applied emission factors for each category are listed in electronic supplementary material, tables ST1 and ST2. Below is a brief description of each category.

National total CH_4_ emission estimates for most emission categories in Australia's National Greenhouse Gas Inventory [29] are publicly available. Most categories are also available at the state level, but a number of CSG-related categories are restricted. In contrast with some other countries (e.g. the USA [30] or the Netherlands [31]), in Australia, there are no publicly available emission estimates for individual facilities for any category. Other sources of publicly available gas and agricultural production data were used to make a verifiable estimate of facility-scale and regional CH_4_ emissions.

For CSG sources, publicly available gas production and produced water data were used, available via the Queensland Government Open Data Portal [32]. These data were used in conjunction with details in environmental impact approval reports, plus satellite images, to collate detailed information on the location and capacity of CSG produced water holding ponds (raw water), wellheads, pipeline lengths, compression stations and gathering and boosting (G&B) facilities. Emission estimates were derived based on these data following the workflow as outlined in Australia's 2018 National Greenhouse Gas Inventory (table 3.44 ‘fugitive emission factors for natural gas' in [29], and sources 1–4 in electronic supplementary material, tables ST1 and ST2). In electronic supplementary material, ST1, a total of nine CSG sources are considered in the estimate: onshore wells (note 1a), an estimate for abandoned onshore wells in the region (1b), venting (1c), flaring (1d), G&B pipeline length (1e), transmission and storage (1f), produced water (2), G&B stations (3) and processing plants (4). Sources 1a–e were distributed proportionally using the wellhead positions. The produced water pond, G&B station and gas plant emissions were located at the latitude and longitude of the facility before being aggregated in the emission grid. In [29], the estimates for G&B stations and gas processing plants are derived from the work by Mitchell *et al*. [33]. The emissions estimated for G&B are calculated using 0.0015 * tonnes of emissions/tonne of gas throughput. The emissions from the gas processing plants (*y*) are derived from *y* = 0.6369 *x*^−0.48^, where *x* is the gas throughput in tonnes. Below, the CSG categories listed above are referred to as the Australian Government workflow for estimating emissions from the CSG sector. The methods and emission factors used by the Australian Government are consistent with international guidelines and are reviewed each year by an international panel of experts. Climatic conditions, farming and industrial practices, in association with societal differences, mean that the emission factors used in this study are often unique to Australia, but consistent within the variability between nations [19,29].

There are four active coal mines (Cameby Downs, Kogan Creek, New Acland and Commodore) and one recently closed coal mine (Wilkie Creek) in the region of the study. These are all open cut mines and emissions estimates for each mine are based on the tonnes of produced coal (electronic supplementary material, table ST1). No emission estimate was determined for the closed coal mine. Other coal-derived sources of CH_4_ in the Surat Basin include ground seeps of unknown origin (either natural or poorly documented coal exploration well sites), and the Condamine River seeps. For these sources, we used the CH_4_ emissions reported in [23].

Agricultural enteric fermentation and manure management CH_4_ emissions for grazing cattle (also called pasture cattle), feedlot cattle and dairy cattle, piggeries and poultry were estimated using Australia's 2018 National Greenhouse Gas Inventory (Volume 1, table 5.12 and 5.17 [29]) emission factors. Animal population statistics for 2018 were sourced from the Australian Bureau of Statistics [34]. The location and animal population data for feedlots, piggeries and poultry point sources were obtained using data from the Queensland Government Open Data Portal [32], Queensland Government Globe [35] and the Farm Transparency Project [36]. All facilities were position-checked using Google Earth [37]. Grazing (pasture) cattle emissions were distributed over the grazing cattle and mixed crops land use regions. In the southeast, there are two major meat works (Beef City and Oakey Beef Exports). These are known sources of CH_4_ emissions [12,20], and an estimate is provided for these facilities based on the number of animals processed and waste volumes. There are 2319 hectares of agricultural ponds in the region. We used the Australian Government estimate for ‘water bodies’ emissions, which were distributed over the Condamine agricultural districts.

Residential emissions estimates were population-proportioned estimates for the Australian Government reported emissions for Queensland [38]. Population statistics for each urban centre were obtained from the Australian Bureau of Statistics [39]. The UNFCCC categories incorporated in the inventory were Energy 1.A.3.b Road Transportation, Energy 1.A.4.b Residential, Waste 5.A Solid Waste Disposal and Waste 5.D.1 Domestic Wastewater.

There are an estimated 487 000 kangaroos in the study area [40] and annually, there is a commercial harvesting. Recent research has demonstrated that kangaroos are a non-negligible source of CH_4_ [41], thus an estimate of kangaroo emissions was incorporated into the region's emissions estimate. These CH_4_ emissions were distributed evenly throughout the forested areas.

### (b) Airborne measurements and top-down flux calculation

#### (i) Airborne measurement platform

The airborne platform used for the Surat flights was a research motorglider owned and operated by ARA (Airborne Research Australia), a Diamond Aircraft HK36TTC ECO-Dimona, equipped with underwing pods carrying the instrumentation, partly from MetAir (Switzerland). One pod carried a Los Gatos Gas Analyser (LGGA) for CH_4_, CO_2_ and H_2_O, together with a pump and an air inlet; the other pod, the MetPOD, carried the meteorological instrumentation (see electronic supplementary material, figure SF4). Sensors in this pod included a three-dimensional high-resolution turbulence probe for air motion (horizontal and vertical wind components), air temperature, humidity and ambient pressure sensors and a modified LiCOR Li-7500 open path gas analyser for fast CO_2_ and H_2_O concentrations. With a cruising speed of 150–170 km h^−1^ and an endurance of more than 5 h, the flight patterns had a total length of about 700 km (more, but not double, with two flights per day).

High-resolution aircraft parameters such as GPS position, altitude, ground- and air-speed, attitude (pitch, roll, aircraft heading) and air-flow angles and accelerations were measured by a combined GPS/IMU system (OXTS RT4003, Oxford Technical Solutions, Oxford, UK). The meteorological parameters were recorded at 20 Hz and aircraft parameters at 250 Hz. The LGGA-output rate was 1 Hz (limited by the available flow rate from the vacuum pump to about 0.5 Hz). Accurate time synchronization for all measurements was ensured by including GPS-generated timestamps in the data streams, and by adjusting any intake delays by comparing, e.g. the slower and delayed H_2_O signal from the LGGA with the fast signal from the Li-7500.

Specifications for the Li-7500 and earlier applications are in [31,42,43] and for the ARA MetPOD on the ARA website [44]. The accuracy, stability and other specifications of an earlier version of the LGGA that was flown by MetAir are discussed in depth in [45,46]. This new LGGA had an internal water vapour correction. More details on the LGGA specifications and calibration checks are presented in electronic supplementary material, supplement J. Accordingly, the stability during the flights was better than 1 ppb. In addition to the continuous measurements, up to 25 discrete bag samples per day were taken for laboratory isotopic analysis (see electronic supplementary material, supplement C).

The accuracy and precision of the measured wind components (*u*, *v*, *w*) from the aircraft are better than 0.5 m s^−1^ and 0.1 m s^−1^, respectively [47–49]. All measured and some relevant derived parameters (such as wind speed and direction) can be monitored on a graphical display in the cockpit, which was essential for adjusting the flight strategy in real time.

The quality of the airborne meteorological measurements (high-resolution three-dimensional wind and turbulence as well as temperature and humidity) is as important as the accurate CH_4_ measurements, because key parameters like the top of the convective boundary layer (CBL, see electronic supplementary material, supplement I), the trajectories with or without diffusion and the density for the conversion from concentrations to column masses depend on them.

#### (ii) Sampling strategy

Based on experience from earlier airborne campaigns with the same type of aircraft and similar instrumentation in other regions and on scales ranging from facility level to regional level [31,50–52], a mass balance approach seemed to be the most appropriate measurement strategy here as well. This required the observation of all mass fluxes in and out of a virtual box standing on the surface of the region of interest, aligned with the wind. The simplest approach is to assume that the fluxes through the two opposite walls perpendicular to the wind, i.e. upwind and downwind of the region (or of a cluster), dominate the balance. The vertical and lateral fluxes through the other three walls can be neglected, while the emissions are injected from the bottom surface. The calculation of these fluxes and the validation of the assumptions is discussed below.

The flight patterns over and around the emission clusters in the southeastern portion of the Surat Basin CSG fields were designed with the aim of quantifying the emissions from one or both clusters, as well as from disaggregated parts of them. ‘Lagrangian flight patterns' aimed to accurately time the upwind and downwind measurements by following the moving air masses along the wind between the upwind-most and thus background transect at the beginning, and one or up to four consecutive downwind transects thereafter. This strategy is schematically shown in electronic supplementary material, figure SF16.

**Figure 2.**
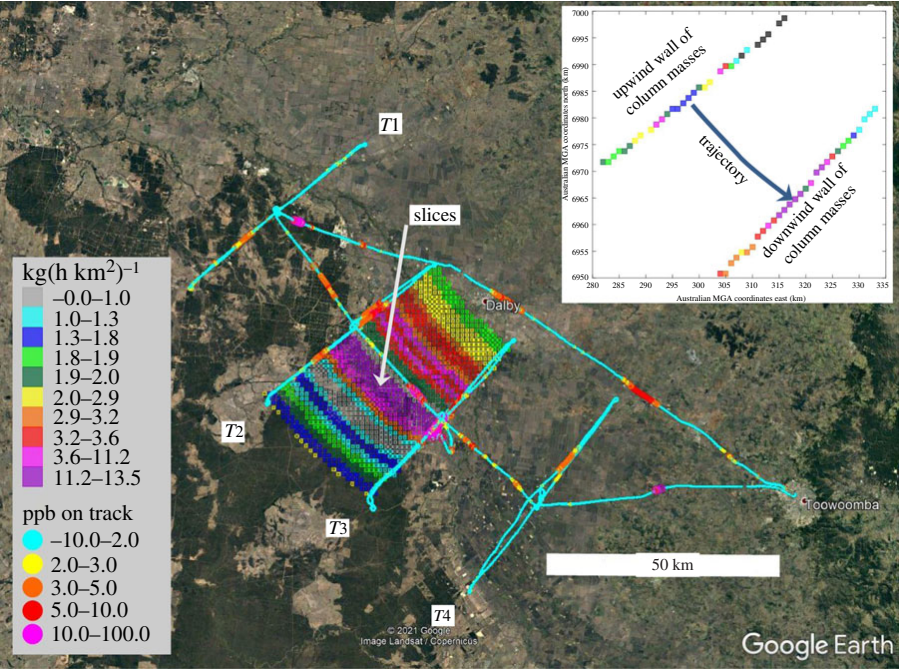
The flight pattern of 15 September 2018, reflecting the Lagrangian flight strategy as shown in electronic supplementary material, figure SF16, for an NW-wind situation. The light blue lines are showing the flight tracks at 150 and 300 m above the ground. The coloured ‘carpet’ consists of slices of 1 km width that are showing the CH_4_ emission estimates along these ‘slices', calculated according to equation (2.1). The emission estimates in kg h^−1^ km^−2^ shown in colour-coding along each slice represent the average emission along a slice, not knowing where along the trajectory (slice), the emissions were picked up. The higher values are aligned with the peak concentrations as indicated on the flight track. The result, as shown as a data point in figure 4 or an entry in electronic supplementary material, table ST4, is the total for the coloured subregion. Other cases are the subregions between *T*1 and *T*4, *T*1 and *T*2, and *T*3 and *T*4. The inset is showing the ‘walls of column masses’ along the transects *T*2 and *T*3 as they are defined by the flight tracks calculated according to equation (2.2) and the explanations in the text and in electronic supplementary material, E. The trajectory connects an upwind pixel with a downwind pixel. If the blue pixel stands for 5 kg of CH_4_ mass (the colour-coding for the kg km^−2^ is different than for the kg h^−1^ km^−2^ or for the ppb on the left), the pink for 15 kg and the travel time was 1 h, then the average emission along the trajectory was 10 kg h^−1^. (Online version in colour.)

To the extent possible, emission fluxes were calculated not only for one given area between the upwind and downwind transects (as in most previous mass balance studies, e.g. [31,51,53]), but these flux estimates were broken down spatially along the transects, thus defining smaller areas within subregions. Referring to figure 2, this means splitting the area along suitable slices, e.g. around a major plume. Then this plume and the areas outside were quantified as individual mass balances. If the mean wind was across the Surat Basin, such splits were particularly useful for the long transects, as shown in electronic supplementary material, figures SF6, SF10 and SF11. More subtleties and how the vertical dimension of the boxes was treated, or how the Lagrangian flight patterns were planned, are discussed in electronic supplementary material, supplements B, H and I.

Of the seven flight days, six were used to calculate mass balances (including four based on precise Lagrangian timing), and the last day was used to inspect sources. Most subregions within the TD domain were visited more than once throughout the six mass balance flights, thereby contributing to multi-day CH_4_ emission estimates of the full TD domain. However, there are no repeat estimates of individual subregions because the flight patterns and orientations within the TD domain changed day-to-day based on wind directions.

As part of this study, 90 air samples were collected and analysed for *δ*^13^C_CH_4__ including a subsample also analysed for *δ*D_CH_4__. The data from these samples will be incorporated using the joint insights from the UNSW inventory and daily dispersion estimates from the atmospheric modelling in this paper. This analysis should provide further insights on source contributions in the southeast portion of the Surat Basin, but is beyond the scope of this paper.

#### (iii) Mass balance flux calculation

The large spatial scale of the surveys in this study requires the assumption of mass conservation between upwind and downwind transects during the measurements because the aerial measurements cannot cover every surface point of the virtual box. However, we validate the assumption as follows. Divergence due to heating of the CBL affects only the mean vertical flux, i.e. the exchange with air above the CBL. The mass conservation principle is justified for meteorological reasons, and deviations from this assumption (a few per cent horizontal divergence or convergence) are acceptable. The details are explained in electronic supplementary material, supplement E, resulting in the two equations (equations (2.1) and (2.2)) applicable to figure 2 or electronic supplementary material, figure SF6, where each transport along a trajectory is defining a ‘slice’ of width ‘*d*’ (1 km in our application) between an upwind and a downwind pixel with an area of *d*^2^

2.1 $$e=\frac{a\times (m_{2}-m_{1})}{t\times d^{2}}=\frac{n\times (m_{2}-m_{1})}{t},\text{ or }\frac{e}{a}=\frac{m_{2}-m_{1}}{t\times d^{2}}$$

and

2.2 $$m=\sum dz_{i}\times M\times \rho_{M}\times c_{i},$$

where ‘*e*’ is the emission rate (kg h^−1^) along a slice, ‘*a*’ is the area of the slice between the up- and downwind pixels, ‘*m*_1_’ is the column mass above the upwind pixel, ‘*m*_2_’ is the column mass above the downwind pixel, ‘*t*’ is the travel time of the air mass and ‘*n*’ is the number of pixels between the up- and downwind transect. The column mass ‘*m*’ (*m*_1_ or *m*_2_) is defined as the sum of CH_4_ mass between the surface and the top of the CBL in the column above each pixel, calculated from the molar density ‘*ρ_M_*’ of the air in each voxel ‘*i*’, the concentration ‘*c*’ (molar fractions above the ‘background concentration’ as explained in electronic supplementary material, supplementJ) measured in each voxel, the molar mass of CH_4_ and the depth ‘*dz*’ of the voxels (50 m in this application).

The above concept of column mass is universal and can, therefore, also be applied to individual plumes (as shown in figure 2 by pink slices). It is also valid when the up- and downwind transects are not Lagrangian, for instance, for air that is moving slower or faster than the sequence of the measurements. This principle holds when the total emissions for a defined region or subregion and the wind field are nearly constant (steady-state conditions). Therefore, we can apply this concept to all flights including the two on 10 and 12 September where the two emission clusters were scanned with a raster pattern perpendicular to the wind, but not in a Lagrangian manner.

The 32 subregions for which we calculated emission rates from all flights were combined to a total regional emission estimate. However, this is not trivial because these subregions overlap. The sum of the emissions from all subregions is, therefore, not the total for the region. As described in more detail in electronic supplementary material, supplement G, BU inventory information about the location of different emission sources within the overlapping subregions was used to estimate the CH_4_ emissions allocated to subregion overlaps.

## 3. Results

### (a) UNSW inventory-based emission estimates (bottom-up)

This research focused on the southeast portion of the Surat Basin CSG fields, including the two CSG clusters shown in figure 1, while all Surat Basin CSG gas production regions were covered by Luhar *et al*. [23]. CH_4_ emissions for the year 2018 were estimated for 25 source categories (electronic supplementary material, tables ST1 and ST2) within a 200 km by 200 km domain (figure 1). The UNSW inventory domain covers a larger area than the aerial measurements (TD domain), but the spatial boundaries were aligned for the TD–BU comparison.

The UNSW inventory CSG CH_4_ emissions are for the production regions itemized in electronic supplementary material, table ST3. Six-monthly tallied gas production data (no condensate is produced) are published by the Queensland Government [16]. To better align gas production with the September 2018 airborne measurements, the gas and water production statistics for the period 1 July 2018–31 December 2018 were doubled to provide the annual estimate for the volume of gas and water produced, 21 500 Mm^3^ and 28 800 Ml, respectively.

**Figure 3.**
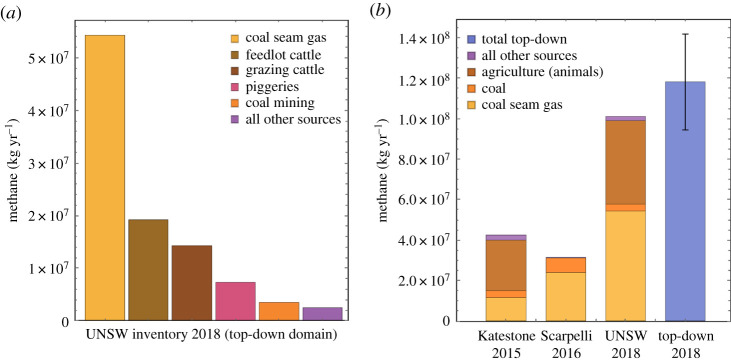
(*a*) Major sources of CH_4_ emissions to the atmosphere in the UNSW inventory (electronic supplementary material, table ST1). (*b*) A comparison of the four CH_4_ emission estimates for the TD domain in the Surat Basin CSG fields (Note: for the Scarpelli 2016 estimate, there are only estimates for CSG and coal). While we use units of kg h^−1^ for the TD estimates throughout this paper, this comparison extrapolates these emissions to annual levels (kg yr^−1^). See text for the contributions of changes in activity over the years versus EFs versus activity data to the emission differences. (Online version in colour.)

Within the TD domain, the UNSW inventory BU estimate for total CH_4_ is 101 Gg yr^−1^, of which 97.6% is released from five categories: CSG, feedlot cattle, grazing cattle, piggeries and coal mining (figure 3*a*). CSG is the largest source of CH_4_, emitting 54.3 Gg yr^−1^ from both production and processing. Cattle are the second largest source of CH_4_, with feedlot cattle producing 19.2 Gg yr^−1^ and grazing cattle 14.3 Gg yr^−1^, for a combined total of 33.5 Gg yr^−1^. Piggeries are the fourth largest CH_4_ source emitting 7.3 Gg yr^−1^ followed by emissions from open cut coal mines, which produce 3.5 Gg yr^−1^. Within the TD domain, all other natural, agricultural, industrial and residential sources of CH_4_ combined only contributed 2.4 Gg in 2018.

Presented in electronic supplementary material, table ST1 is the Katestone BU inventory estimated to provide a prior for the Bayesian inverse framework presented in [23]. This inventory was for the year 2015 and covers an area of 350 km by 350 km. This encompasses the complete Surat Basin CSG production region and an extensive area of cattle production to the west and northwest of the UNSW inventory. The Katestone inventory overlaps with the UNSW inventory for the southeast quadrant. Within the TD domain, the Katestone inventory estimate for annual CH_4_ emissions from all sources is 42.5 Gg yr^−1^, with 11.5 Gg yr^−1^ from CSG sources. The Katestone inventory estimates CH_4_ emissions of 17.2 Gg yr^−1^ for feedlot cattle, and 7.1 Gg yr^−1^ for grazing cattle. The open cut mines are estimated to emit 3.5 Gg yr^−1^ and the piggeries 0.6 Gg yr^−1^. Within the TD domain, the Katestone CH_4_ emission estimate for CSG, cattle and piggeries is lower than the UNSW inventory estimate by about 370%, 40% and 1100%, respectively.

Within the TD region, a portion of the total CH_4_ emission estimate difference between the Katestone and UNSW inventories is due to increased emissions from animals: 25 Gg yr^−1^ (2015) versus 41 Gg yr^−1^ (2018). This is due to the growth of the cattle and pig numbers in the region, in part due to the agricultural sector establishing new facilities near the CSG produced water. Between 2015 and 2018, gas production increased from 12 434 to 21 500 Mm^3^ (a 73% increase). However, the Katestone CH_4_ emission estimate is only 11.5 Gg yr^−1^, while the UNSW CH_4_ emission estimate is 54 Gg yr^−1^ (370% larger). Based on the Australian Government workflow used for the UNSW inventory, the Katestone inventory has underestimated emissions. While previous inverse tower modelling [23] suggests that the Katestone inventory underestimates CSG emissions by 33%, our study suggests that this underestimation is more severe.

There is considerable uncertainty associated with BU inventory estimates, but it is difficult to quantify due to a lack of data. For example, the government departments do not report the errors associated with animal counts nor the volume of gas produced. We know the total cattle count for the year, but we do not know on any day how many cattle are in the feedlots, or in the paddocks (or eating grass along the roadside as allowed in the region). Numerically simulating the impact of the uncertainty is beyond the scope of this project.

### (b) Aerial measurement-based flux estimates (top-down)

**Figure 4.**
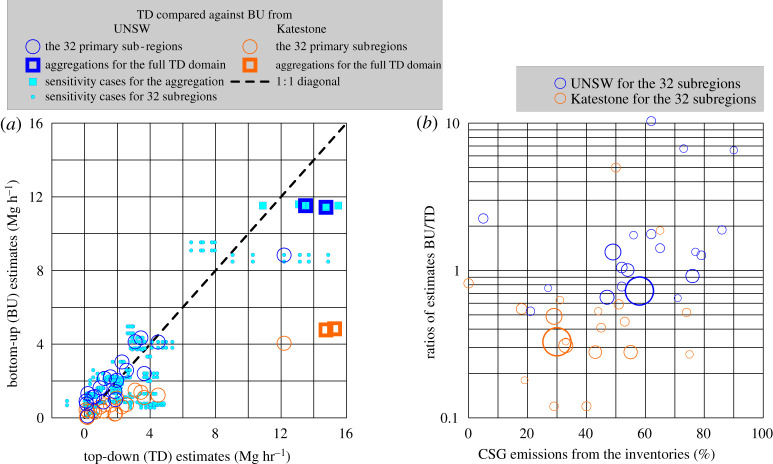
Summary of all mass balances. (*a*) BU versus TD for the individual mass balances (circles) and for two subregional aggregations (squares) compared with the UNSW and the Katestone inventories. The light blue points and squares are showing the different sensitivity cases as explained in electronic supplementary material, I and K and listed there in electronic supplementary material, tables ST5 and ST6. These parameter variations are primarily influencing the TD results, but indirectly BU as well (see caption to electronic supplementary material, ST5). (*b*) The BU/TD ratios according to the UNSW and Katestone inventories as a function of their CSG percentage. The diameters of the circles are proportional to the areas of the mass balances. (Online version in colour.)

Based on six days of flights dedicated to regional and subregional mass balances, 32 overlapping mass balances and hence TD emission estimates were calculated, displayed in figure 4 and listed in electronic supplementary material, tables ST4–ST6. The flight patterns of the six days are presented in detail in the supplement (electronic supplementary material, figures SF7–SF12). It was possible to observe three wind regimes, i.e. one direction along the Surat Basin (NW wind), and both directions crossing the basin (SSW, and NNE). On four days, the air masses picking up CH_4_ emissions were observed by nearly perfect Lagrangian flight patterns, while the first two days (10th and 12th) mapped the two main emission clusters during weak southerly and weak northerly winds, respectively. Assuming constant total emissions within each estimated subregion, i.e. accounting for mutually offsetting effects of potential temporal variability of individual emitters within a subregion, and considering the observed near constant wind, the emission estimates from these two flights are as good as the other ones for emission estimates using the method described above.

The subregions between the up- and downwind legs along the basin for wind across the basin (16 and 18 September) were longer than the legs for wind along the basin, capturing both CSG clusters. Therefore, the total mass balance for 16 September in electronic supplementary material, figure SF6 covered almost the whole TD domain. As described in the Methods section, such large areas were split into subregions chosen in a way that each subregion contained some groups of sources. Three examples of such 'sub-sub-regions’ are explained in electronic supplementary material, figure SF6. All individual 32 overlapping subregions with their colour-coded ‘slices' are provided as kmz files in electronic supplementary material, supplement F. The sum of the subregions between the long transects visible in electronic supplementary material, figures SF6 and SF10 (bal_16_1 and bal_16_2) covers almost the whole TD domain and, therefore, represents a first estimate for the total emission of both CSG clusters and their surroundings. According to figure 4 and electronic supplementary material, table ST6, this is a CH_4_ emission rate of 14.7 Mg h^−1^, whereas the aggregation of all the subregions, including a sensitivity study, estimates the total emission rate to be between 10.9 and 15.5 Mg h^−1^ (reference case and median 13.5 Mg h^−1^).

The scatterplot in figure 4*a* and the values in electronic supplementary material, tables ST4 and ST5 show the TD emission estimates and their uncertainties for the individual subregions. The quantified TD flux uncertainties include the mixing height (zCBL as shown in electronic supplementary material, figures SF19 and SF20) and the associated changes in the background concentration caused by entrainment from above the CBL as well as wind measurements. These uncertainties were assessed by sensitivity analyses varying the critical parameters within wide ranges. According to electronic supplementary material, tables ST5 and ST6, the resulting uncertainty for the total emission estimate for the study region is about 16%. However, since the homogeneity of background concentrations and all considerations discussed in electronic supplementary material, supplement N cannot be validated in every detail, we report here a conservative uncertainty estimate of 20%. This conservative estimate also accounts for other minor sources of error including the precision of CH_4_ concentration measurements.

The TD estimates are all compared against BU estimates for the same subregions or combinations thereof (electronic supplementary material, ST6) from (i) the spatially resolved emission inventory developed here (UNSW, previously defined), and (ii) the existing Katestone gridded emission inventory [23]. The two aggregated results are: (i) the regional mass balance from 16 September, which is fully independent of the inventory, and (ii) the combined estimate of multiple subregional mass balances after accounting for overlapping subregions based on UNSW inventory information about the location of different emission sources (see Methods; electronic supplementary material, supplement G).

### (c) Comparison of bottom-up and top-down flux estimates

The scatter plot in figure 4*a* compares the UNSW and Katestone BU datasets with the TD emission estimates for the 32 subregions. The two aggregated results for the TD domain are depicted as squares. Electronic supplementary material, table ST4 shows that typical CH_4_ emissions in the area are about 1–5 kg h^−1^ km^−2^.

**Figure 5.**
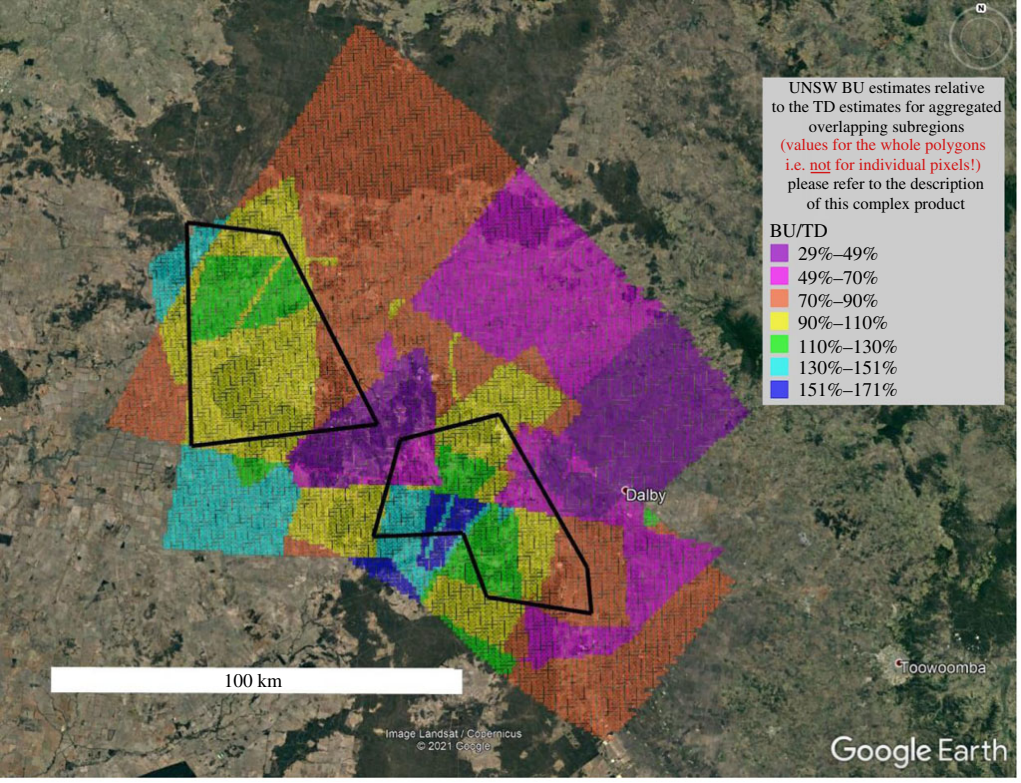
This ‘patchwork’ covering exactly the TD domain shows the percentage of BU (UNSW) versus TD emission estimates according to the mass balances. The two CSG clusters are represented by the black polygons. The yellow areas, e.g. indicate that on average, all emissions from the inventory in this coloured area were reflecting 90–110% of the TD estimates for the same areas. These percentages are not valid for the individual pixels, but for the connected areas with the same colour only. They are also influenced by all the overlapping subregions defining a monochrome polygon. For the pure individual mass balances of the 32 overlapping subregions and their uncertainties, refer to electronic supplementary material, tables ST4 and ST5. Different sensitivity scenarios for the emissions within the coloured full TD domain are listed in electronic supplementary material, table ST6. (Online version in colour.)

Figure 4*b* shows the ratios of BU/TD emission estimates against the portion of CSG emissions as a percentage of total emissions in the UNSW inventory for the TD domain. The diameters of the circles are proportional to the areas of the subregions, i.e. the positive outliers represent small regions, where the inventory strongly overestimates the TD emission estimates. The UNSW inventory estimate accounts for 75–105% of aggregated TD estimate (i.e. the inventory and TD estimate converge within the TD uncertainties). By contrast, the Katestone BU estimate [23] misses over half of the observed total emissions from all sources (details in electronic supplementary material, table ST6). As described in the Discussion, for CSG sources about half of this underestimation is due to underestimated emissions, while the other half is due to lower gas production in 2015 (12 434 Mm^3^) compared to 2018 (21 500 Mm^3^). Despite the slight underestimation in the UNSW inventory, figure 4*b* suggests that the source attribution between CSG and non-CSG sources according to the UNSW inventory is generally realistic because, for example, a systematic BU overestimation of CSG sources would cause a positive correlation in this point cloud, and vice versa. The same is true for the Katestone inventory, which, however, strongly underestimates total emissions as discussed above. Figure 5 indicates how the UNSW BU under- and overestimations are distributed spatially across the study area.

Given the generally close agreement between the TD and UNSW BU estimates for the majority of subregions, any adjustments to (i) BU emission factors or (ii) spatial allocation (e.g. feedlot to grazing) would be relatively small. Nevertheless, all cases with a BU/TD ratio less than 50% or greater than 150% (i.e. no BU and TD agreement within ±50%) were inspected individually to derive insights on candidate sources for revisions and iterations throughout the development of the UNSW inventory. These cases are listed below. Below, we use a threshold of 20% of the subregion CH_4_ emissions for isolating significant sources within a subregion. This was selected based on the distribution of points in electronic supplementary material, figure SF27.

In the subregions where the BU estimates exceed the TD estimates, the CSG sources dominate CH_4_ emissions (66% of total CH_4_ emissions according to the UNSW inventory). This suggests that the CSG emissions might be slightly overestimated in the UNSW inventory. G&B stations are the biggest contributor to CSG emissions in the overestimated subregions (subdomains 10_02, 10_03, 15_02,16_04, 18_02, 18_05 and 18_06 electronic supplementary material, figure SF27). However, the evidence is not unequivocal.

The feedlot cattle are the second largest contributor to emissions in the BU overestimated subregions (subdomains 12_02 and 15_02), and the UNSW inventory assumes that they operate at full capacity. Reducing the feedlot cattle population to a percentage of the licenced capacity would improve the match between BU and TD emission estimates in these subregions. In fact, removing feedlot emissions from the subregions would in part reduce the need to decrease CSG emissions in order to match BU and TD estimates. There are two subdomains where piggeries contribute greater than 20% of the CH_4_ emissions (15_04 and 15_05); these sources require closer examination. There are also cases where the CSG water ponds (10_03, 16_04, and 19_01) contribute greater than 20% of the subregion emissions. In subregion 18_4 coal mining contributed greater than 20% of emissions, so at this location, the total emission estimate would be sensitive to changes in the emission estimate for coal.

In the subregions where the TD estimates exceed the BU (BU/TD 0–50%, electronic supplementary material, figure SF27), there are four subdomains where CSG G&B contribute greater than 20% of the subregion emissions (16_09, 16_10, 19_03, and 19_04). This highlights that generalizations about changing emission factors for the CSG G&B stations must be treated with caution and examined more closely with targeted studies.

In the subdomains where BU/TD ranged from 0 to 50% (figures 1 and 5, electronic supplementary material, figure SF27), there were two subdomains where feedlots contributed greater than 20% of the emissions (16_10 and 19_04) and one domain where piggeries were a dominant source (16_09). It is not possible to tell from these data if these major sources in these subregions are being underestimated, or if some other category is being underestimated. There are also other unaccounted sources in the region that need close inspection before any adjustments are made to the emission factors. Sources not incorporated into the UNSW inventory that are located in the underestimated regions include CH_4_ emissions from the use of town biosolids as fertilizer, additional natural gas seeps and a closed coal gasification site south of Chinchilla (operated by Linc Energy).

Since all individual CH_4_ sources are unlikely to be constant over time, the scatter in the BU/TD ratios across subregions in figure 4 is expected because relatively few individual sources are located in each of the 32 subregions. However, our results demonstrate, similar to a previous study [50], that such scatter is expected to decrease over the full TD domain as temporal variations tend to average out across subregions. Note that the full regional mass balance on 16 September is in good agreement with the multi-day flux aggregation of subregions, which is further evidence that day-to-day as well as subregional flux variability is well accounted for by our aerial measurements.

## 4. Discussion

### (a) Benefits of spatially resolved top-down and bottom-up estimates

The data collection approach and analytical methods in this study were designed to address the gaps in the understanding of CSG-related CH_4_ emissions from previous studies. Specifically, this includes a comprehensive sampling of all CSG-related facilities beyond gas wells [21,22] such as gathering compressor stations, processing facilities and water ponds by means of the TD regional and subregional mass balances. Further, instead of relying on a prior estimate [23], the aerial-based survey in this study estimates total regional CH_4_ emissions independently. In this study, the CSG industry portion of the total regional CH_4_ emissions is based on the UNSW inventory. However, the total emissions and the source attribution of this spatially resolved inventory are validated via the aerial survey. As described above, the spatially resolved TD estimate thus reinforces the BU estimate rather than depending on it.

The spatial disaggregation of the total TD CH_4_ emission estimates is a methodological advancement relative to most previous studies (e.g. [51–54]), which report a single regional emission total. The spatial disaggregation was achieved by (i) Lagrangian flight paths dissecting the TD domain into subregions, and (ii) further dividing the subregions into groups of slices along the trajectories. Apart from yielding more detailed spatial information about the total emissions, this approach has multiple advantages. First, the Lagrangian planning of the flight paths ensures that the precise air mass measured upwind is measured again downwind, thus avoiding the assumption of a constant CH_4_ background over time. Second, emission estimates are more precisely defined to a specific region, which is especially important in a large region like the Surat Basin where CH_4_ emitters extend far beyond (both upwind and downwind) of the region studied. Third, approximate locations of individual large emitters (along a slice, or a group of slices) can be identified (see examples in figure 2, e.g. purple slices, or electronic supplementary material, figure SF6).

Expanding on the last item, in some cases, individual large plumes along a transect can be isolated to estimate CH_4_ emission rates from an individual source. An example of a single source amounting to 2.7 Mg h^−1^ is described in electronic supplementary material, supplement P. Even without precisely identifying this CH_4_ emitter, it became clear that an unknown source, or a known source with enhanced emissions, is within the pink area northeast of Dalby in figure 5. Both the structured regional emission pattern on such maps, and the individual plumes, helped to update the UNSW emission inventory throughout this study in an iterative manner. Specifically, the aerial surveys highlighted the requirement to add source categories (meat works, kangaroos and on-farm water bodies) and review the feedlot location data. Additional research, either a numerical analysis of this dataset or more detailed field measurements, is needed to quantitatively evaluate the impact of further adjustments to the UNSW inventory emission factors in the under- or overestimated subregions.

### (b) Methane loss as a fraction of coal seam gas production

The gas production normalized emission rate (PNER), colloquially referred to as CH_4_ loss rate, is a common metric for comparing O&G-related CH_4_ emissions across regions as it accounts for differences in production volumes (over time and space) and extraction methods. In this case, for example, the Katestone inventory was reported for absolute emissions and production during the year 2015 while our measurements occurred during 2018. Apart from scientific studies, the metric is also used by O&G operators to publicly report their current ‘CH_4_ intensities’ as well as future targets [55]. Here, we compare the annual PNER in the Surat Basin based on the TD-validated UNSW inventory with other estimates for the same region as well as with those across other regions. Summarizing the above, the UNSW inventory PNER is 0.37% of the production volume, which is 2.64 times greater than the 0.14% determined using the Katestone inventory, and 2.07 times greater than the 0.18% in Scarpelli *et al*. [56]. In the example of the Katestone inventory, the PNER difference explains almost half of the absolute CSG emission differences between the Katestone 2015 estimate and the UNSW 2018 estimate. Considering an annual CSG production of 20 000 Mm^3^, the amount of CH_4_ emitted to the atmosphere for PNER values of 0.14% and 0.37% are 19 Gg yr^−1^ and 50 Gg yr^−1^, respectively.

**Figure 6.**
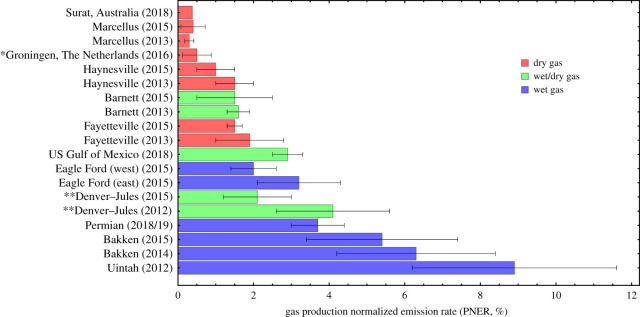
Percentage of produced gas emitted to the atmosphere for oil and gas fields in the USA [50–54,57–61], The Netherlands [31] and the Surat Basin, Australia (TD-validated estimate of the discrete UNSW inventory). *All literature values are pure TD quantifications except for the Groningen field in The Netherlands where Yacovitch *et al*. [31] reported only the TD measurement-based absolute emission value. The upper bound PNER shown here divides this TD estimate by the gas produced in the same region [62], but this is an overestimate because it does not account for emissions from gas transport and distribution that is produced elsewhere. The lower bound PNER shown here is a BU estimate using Dutch government data [63]. The bar represents the mean value between TD and BU. **The Denver–Jules Basin includes both oil wells and gas wells, but production infrastructure is heavily dominated by oil [59]; the basin may, therefore, also be characterized as a wet gas basin. (Online version in colour.)

As illustrated in figure 6 (and references therein), CH_4_ emissions and PNER for the major O&G production basins in the USA are highly variable, with TD estimates ranging from 0.3 to 9% (mostly based on aerial surveys, but also a multi-species tall tower study and one combined aerial/satellite study). The 0.37% is comparable with (i) the lowest reported PNER in the USA, i.e. 0.3–0.4% in the dry gas portion of the Marcellus Shale and (ii) the 0.5% reported in the Groningen onshore field in The Netherlands [31]. There are a number of plausible reasons for the relatively low PNER from CSG production in the Surat Basin compared to other US regions. Generally speaking, as indicated by the colouring in figure 6, the empirical data suggest that dry gas basins (almost no oil/condensate production) tend to have lower PNER than wet gas or mixed basins (co-production of oil and gas), likely in part due to the lack of (i) oil/condensate tanks and (ii) processing needed to separate O&G at the well pad [64,65]. Nevertheless, the PNER may also depend on other, sometimes interconnected factors including complexity of gas infrastructure and production processes, separation and storage of water, well productivity, extent of venting and flaring, equipment age, gas network design, gas chemistry, maintenance schedules and geological settings.

### (c) Impact of data choices in inventories

The UNSW inventory was developed based on local knowledge of the existing CH_4_ source categories and data sources and in-depth research of the appropriate emission factors. Additionally, the aerial survey helped identify CH_4_ sources that were not initially listed in the inventory (see the previous section). As a result, the UNSW inventory matches the TD estimates relatively well even though the UNSW inventory still slightly underestimates the measured TD CH_4_ emissions. The importance of the careful selection of emission factors and the comprehensive incorporation of all CH_4_ sources during inventory development is illustrated in a comparison with other inventories for the same region.

Figure 3*b* compares the total CH_4_ emission estimates of the TD approach and the UNSW inventory with the inventories by Katestone [23] and Scarpelli *et al*. [56]. The Katestone inventory supplied for this study is a regional gridded (5 km by 5 km cells) inventory for all CH_4_ sources, while the Scarpelli *et al*. inventory is a global gridded (about 11 × 11 km) inventory for oil, gas and coal CH_4_ emissions only, which was derived by spatially redistributing the national emissions reported to the UNFCCC. The comparison further includes the source attribution of the total CH_4_ emissions into CSG, coal mines, as well as (except for Scarpelli *et al*.) agriculture and remaining sources combined (other). Gas production increased between each study: 12 433 Mm^3^ (2015), 19 743 Mm^3^ (2016) and 21 500 Mm^3^ (2018), which accounts for about half of the variation in reported CH_4_ emissions in figure 3*b*. But if the PNER of 0.37% established in this study is correct, then both the Katestone and Scarpelli *et al*. workflows have resulted in an underestimation of emissions.

Only the UNSW inventory is verified using an independent TD estimate. Using the UNSW PNER:

— in 2015, the CSG emission estimate is 31 Gg yr^−1^, not 11 Gg yr^−1^ as reported by Luhar *et al*. [23], an underestimation by 20 Gg yr^−1^.
— in 2016, the CSG emission estimate is 49 Gg yr^−1^, not 24 Gg yr^−1^ as reported by Scarpelli *et al*. [56], an underestimation by 25 Gg yr^−1^.

These large underestimations highlight the need for independent, empirically based estimation of emissions for comparison with inventories, which are subject to errors with source data, emission factors, spatial allocation and temporal scaling. The precise reasons for the large underestimations in the Katestone and Scarpelli *et al*. inventories are not clear, but figure 3*b* illustrates that available choices in inventory collation result in considerable uncertainties. The side-by-side comparison highlights the need for improvements using empirical data as witnessed in numerous previous studies (e.g. [28,31,66,67]).

### (d) Outlook

There are no comparable studies on CSG production regions in the USA or other locations worldwide. While CSG production in the Surat Basin likely shares certain dry gas characteristics with the Marcellus Shale, Haynesville and Fayetteville basins, further work is required to understand the reasons for the relatively low PNER in the Surat Basin compared to these locations.

As in the previous TD studies listed above, the TD estimates in this study are snapshots over several days in one month during 2018, while the inventory estimates are annualized. Studies in three previous locations, i.e. the Four Corners region in the USA [26,68], the Permian Basin in the USA [60], and a region in Mexico [67], used satellite-based long-term measurements to confirm the TD estimates of the local, more spatially detailed, short-term aerial-based surveys. Further research may revisit the Surat Basin using satellite-based analysis, especially considering the ongoing development in remote sensing technology, or via repeated aerial surveys including facility-level measurements to address the open questions about, e.g. emissions factors for feedlots, grazing cattle and G&B stations and potential seasonal variations. A measurement-based investigation about G&B stations is currently ongoing in Queensland [69]. A facility-level airborne study with additional flights by this team using the same measurement tools and similar strategies is currently underway in the Surat Basin and in NW-Western Australia.

The ground-based CH_4_ isotope data reported in [20] demonstrate that *δ*^13^C_CH_4__ measurements can be used to differentiate between CSG and other CH_4_ sources in the Surat Basin only when the data are interpreted in spatial context and prevailing weather data. There is also potential to use both *δ*^13^C_CH_4__ and *δ*D_CH_4__ source signatures to separate CSG and cattle emissions from other CH_4_ sources in the region [20]. As shown throughout this study, the detailed inventory and the high-resolution weather data collected during the flights provided the required controls to interpret both the *δ*^13^C_CH_4__ measurements and the dual *δ*^13^C_CH_4__ and *δ*D_CH_4__ data. The inventory for the blended isotopic value for upwind sources should match the downwind isotopic chemistry of the air collected in the bag samples. This is possible if the isotopic signature of each source has been well characterized and the proportional rates of each source in the BU inventory have been accurately quantified. It is envisaged that these analyses will enable the verification and refinement of inventories.

## 5. Conclusion

This study contributes to international efforts to measure CH_4_ emissions in the O&G and other sectors to help companies and governments prioritize mitigation actions and policies. Here, we present results of the first comprehensive aerial measurement campaign of a CSG field worldwide, the Surat Basin in Queensland, Australia. The objectives were to quantify CH_4_ emissions from all major sources based on TD and BU approaches, and to reconcile any differences. This was achieved via the following combination. First, employing and further developing established aerial surveying techniques to derive spatially resolved CH_4_ fluxes. Second, compiling a spatially resolved CH_4_ inventory based on local insights on CH_4_ emitters and data sources, applying Australia's UNFCCC reporting workflow, and iterating to incorporate missed CH_4_ sources as identified by the aerial survey. The spatial resolution of this present aerial study is a crucial complement to the existing two-tower-based inverse study [23]. It is important to note that the inverse study [23] estimates the Surat Basin total CH_4_ emissions correctly within approximately 10% of Australia's UNFCCC reporting workflow [69]. However, the inverse study's source attribution suffers from its dependence on the Katestone inventory as a prior, resulting in an upward revision of the Katestone CSG CH_4_ emission estimate of only 33%. Our study shows that this value should be approximately 170%.

Our TD–BU reconciliation indicates that CSG sources emit about 0.4% of the produced gas, which is two to three times greater than existing inventories for the region [23,50]. This relative CH_4_ loss is comparable to two other TD measured onshore dry gas production regions, i.e. the Marcellus Shale in the USA and the Groningen field in The Netherlands [31,51,54]. While two other US dry gas basins (Fayetteville and Haynesville) emit more (about 1–2% of the produced gas [50,51]), basins with significant oil production emit substantially more (about 2–9% [52,57,60]). The lower relative emissions in dry gas basins (including the Surat Basin) are likely a combination of larger amounts of gas produced (PNER denominator) and less complexity of gas infrastructure resulting in fewer emissions (PNER numerator).

The workflow for Australia's UNFCCC CH_4_ emissions reporting for the CSG sector is partly based on empirical data in the USA, and it was used in the BU inventory developed here. Our TD–BU reconciliation suggests that our workflow represents observed emissions more realistically than existing inventories for the Surat Basin [23,56]. Plausible candidates for further improving our BU inventory were identified, which need further research. These include an upward revision of the grazing cattle emission factor to better represent Queensland's conditions, and possibly reducing the emission factor for G&B stations. Feedlot emissions estimates also need reviewing. Daily, weekly or monthly gas throughput data would result in better estimates of CH_4_ emissions from CSG facilities and would enable better comparison with airborne measurements. Our study illustrates that considerable advancements in global carbon accounting could be achieved by incorporating independent TD greenhouse gas verification surveys, including the aerial surveys presented here, into national reporting.

## Data accessibility

The datasets supporting this article are mentioned in the electronic supplementary material, section F.

The data are provided in electronic supplementary material [70].
